# QUALZICE: A QUALitative exploration of the experiences of the participants from the ZICE clinical trial (metastatic breast cancer) receiving intravenous or oral bisphosphonates

**DOI:** 10.1186/1745-6215-14-325

**Published:** 2013-10-09

**Authors:** Annmarie Nelson, Debbie Fenlon, Jenny Morris, Cathy Sampson, Emily Harrop, Nick Murray, Duncan Wheatley, Kerenza Hood, Gareth Griffiths, Peter Barrett-Lee

**Affiliations:** 1 Marie Curie Palliative Care Research Centre, Wales Cancer Trials Unit, Cardiff University School of Medicine, Cardiff, UK; 2 Faculty of Health Sciences, University of Southampton, Southampton, UK; 3 Faculty of Health, Education and Society, University of Plymouth, Plymouth, UK; 4 North Adelaide Oncology, Calvary North Adelaide Hospital, North Adelaide, Australia; 5 Royal Cornwall Hospitals NHS Trust, Truro, UK; 6 South East Wales Trials Unit, Cardiff University School of Medicine, Cardiff, UK; 7 Wales Cancer Trials Unit, Cardiff University School of Medicine, Cardiff, UK; 8 Velindre Cancer Care, Cardiff, UK

**Keywords:** Breast cancer, Bisphosphonates, Patient experience, Qualitative, Clinical trial

## Abstract

**Background:**

This qualitative sub-study aimed to explore the experiences of participants on the National Cancer Research Institute ZICE clinical trial, a randomised trial assessing two types of bisphosphonate treatment in breast cancer patients with bone metastases. Participants in the clinical trial were randomly allocated to receive either zoledronate, delivered by an intravenous (IV) infusion at clinic, or oral ibandronate, taken at home.

**Methods:**

Qualitative research interviews were conducted with participant groups organised by treatment and location. Interviews covered experiences and understanding of bisphosphonate treatment, the experience of the delivery mechanisms (IV or oral), side effects and benefits, and quality of life issues. The analytic framework was interpretative phenomenological analysis.

**Results:**

This paper reports on one of four superordinate themes: participants’ experience of the ZICE trial, which explores the participants’ experiences with clinical trial-related processes. Results show that participants were generally satisfied with their randomised treatment, although most participants had an initial preference for oral bisphosphonates. Some difficulties were reported from participants for both interventions: needle phobia, poor veins, difficulty with swallowing and gastric side effects, but pain control was improved with both modes of delivery. However, the infused bisphosphonate was reported to lose effectiveness after three weeks for some participants, whereas the oral bisphosphonate was reported to give consistent pain control. Geographical location and distance to travel made little difference to convenience of access to clinic as the reported lengths of travel time were similar due to traffic congestion in the urban areas. Most participants understood the trial processes, such as randomisation, and information about bisphosphonates but some participants showed little understanding of certain aspects of the trial. Some participants reported difficulties in accessing dental treatment due to their dentist’s perceptions of bisphosphonate treatment.

**Conclusions:**

In trials of medicinal products, especially when testing for non-inferiority, participants’ preferences and idiosyncrasies in relation to treatments should not be assumed. This study has shown that in a trial context, participants’ views can usefully add to the main trial outcomes and they should be taken into account when prescribing in the real world.

**Trial registration:**

ISRCTN13914201. Main ZICE MREC: 05/MRE09/57. CRUK E/04/022.

## Background

The QUALZICE study is a multicentre, qualitative sub-study that explores the experiences of participants in the National Cancer Research Institute (NCRI) randomised phase III ZICE trial, summarised in italics below:

*The ZICE trial is a randomised trial*, *sponsored by Velindre NHS Trust*, *assessing two types of bisphosphonate treatments in breast cancer participants with bone metastases. Participants are randomly allocated to receive either zoledronate*, *delivered by an intravenous (IV) infusion at clinic*, *or oral ibandronate*, *which is taken at home. Zoledronate is considered*, *in many centres*, *to be the standard bisphosphonate of choice*, *given as an IV infusion over 15 minutes every 3–4 weeks. Oral ibandronate could potentially have significant advantages over IV zoledronate as the tablet is small*, *taken once daily at home and without the side effects or inconveniences of an IV infusion. However*, *a large scale direct comparison between IV zoledronate and oral ibandronate has not been carried out and ZICE aims to address this with the enrolment of 1400 participants as a non-inferiority trial using a primary endpoint of skeletal related events. The trial was coordinated by the Wales Cancer Trials Unit and developed on behalf of the NCRI Breast Cancer Clinical Studies Group*^*a*^.

When comparing these two treatments within the clinical trial, in addition to looking at the outcomes of the trial (that is, treatment efficacy, safety, health economics and quality of life), it is important to consider the experiences of participants receiving either drug and how they understand the procedures associated with the trial.

Quantitative data from the clinical trial will provide information about safety and efficacy aspects, as well as an analysis of quality of life and the cost of treatments, using questionnaires. These quantitative comparisons will be used to determine the treatment that should become routine treatment for patients. This qualitative study adds an in-depth, participant-led exploration of individual experience of the treatments and participation in the clinical trial, which cannot be collected using questionnaires.

Qualitative methodologies are commonly used to explore the understanding and experiences of patients and health professionals in health and illness research and are well established in mixed-method evaluations of complex public health or service interventions, which increasingly incorporate process evaluations as an integral part of randomised control trial designs [1-3]. Historically, although the importance of patient experience is recognized to an extent in clinical investigational medicinal product (IMP) trials, efforts to capture such impacts have been restricted to quality-of-life tools and other quantitative scales.

Qualitative approaches provide an opportunity to generate more detailed and in-depth insights into patient experiences than cannot be captured by standardized tools [4]. As such, the role of qualitative research in clinical trials is also growing. For example, there are a number of empirical examples that demonstrate how qualitative research can improve the design and running of clinical trials [5,6].

Reporting on the results of the QUALZICE study, this paper demonstrates that patient experiences can be explored in depth, and that embedded qualitative methods can be used in clinical trials alongside quantitative measures of participant outcomes to generate insights that are of interest for ethical as well as more practical reasons, and that could inform future trial design and implementation. At the request of the chief investigator and the ZICE Trial Management Group (TMG), this paper is written in a style intended to be accessible to a broad audience and is designed to engage with triallists and clinicians.

The aims of the QUALZICE study were to understand the experiences of people taking part in a clinical trial of a supportive care intervention and to explore the different experiences between taking a supportive care intervention by IV infusion or orally.

## Methods

The QUALZICE study was developed on behalf of the ZICE TMG (which includes clinicians, methodologists and patient representatives). It was funded by Velindre NHS Trust’s small grants committee and was coordinated by the Wales Cancer Trials Unit (WCTU). The study was supported by Cancer Research UK and Marie Curie core-funded staff at WCTU. The protocol received the favourable opinion of the Cambridgeshire 4 Research Ethics Committee.

### Recruitment

Participants were recruited from three locations in England and Wales, which vary in their urban or rural characteristics, to give a range of participant experiences. A key difference between the administration of oral versus IV therapy is the need for the participant to attend the centre where the IV infusion is delivered. It was therefore thought that ease of access to the medical centre would affect the patient experience and so participants were purposively selected from the three sites.

Sites were selected purposively and pragmatically if they were a ZICE recruiting site, were able to demonstrate a spread of urban and rural characteristics and were local to a suitably skilled researcher.

Sampling of participants was purposive, aiming to engage homogeneous groups in exploring recurring themes. The participants were categorised according to the treatment received (IV infusion or tablet) and also by type of location (semi-urban, rural or urban). Therefore, overall from the three centres, there were six groups.

We aimed to recruit six to ten participants to each group in line with the usual recommendations for sample sizes when applying interpretative phenomenological analysis (IPA) methodology. The justification for this sample size per group is well supported in the methodological literature with the intention of depth, rather than breadth of analysis [7]. From the 6 groups, a total of 42 patients were interviewed. Data collection ceased when a representative sample had been recruited to each group. IPA methodology is highly descriptive and not reliant on saturation of data, neither is it intended to be generalisable across populations [7]. The numbers of participants in each group are given in Table 1.

**Table 1:** The number of participants, where they lived and their trial arm

	**Semi-urban**	**Rural**	**Urban**	**Total**
Infusion	6	9	6	21
Oral	3	8	10	21
Total	9	17	16	42

Participants meeting the following criteria were included in the study:

1. ZICE clinical trial participants (breast cancer participants with newly diagnosed bone metastases)

2. had been receiving their allocated ZICE protocol for a minimum of 12 weeks

3. were able and willing to give informed consent to participate in the study and to discuss issues relating to their diagnosis, treatment and quality of life issues

4. were able to understand questions and speak English to the extent needed to participate in the interview

Participants were not included in the study if there was any factor that affected communication or comprehension. Eligible participants from the ZICE trial were approached by the research nurses at the recruiting site, where they were given study information to see whether they would like to enter QUALZICE. At their next visit, if they agreed, their contact details were passed to the researcher to arrange an appointment. Written consent was taken at the time of the interview by the researcher.

### Data collection

Individual semi-structured interviews were undertaken with participants at a time and location of their choice. The interviews were up to 52 minutes in duration, and the mean interview time was 22 minutes. All but two of participants chose to be interviewed at home.

The study team developed a master interview schedule with questions and prompts; however, the interviews evolved over time with the interview content developing in line with the interviewees’ answers. A second version was produced after the first few interviews and was used for the remainder of the study. Smith and Osborn [8] highlight the importance of interview schedules being used to guide rather than dictate the interview, enabling participants to discuss issues pertinent to them. The interview schedule was therefore used to ensure that the interviews gathered the required information for the trial, while allowing participants the space to speak about wider concerns. Interview questions are outlined below.

Version 1:

a) Which treatment are you on?

b) What is your understanding of why you are taking this treatment?

c) Take us through what happened at your last visit to clinic?

d) What do you have to do at home for your treatment?

e) How has the treatment affected your daily life?

f) Do you have any new symptoms since taking the treatment?

g) Does your treatment affect your family/social life?

h) Is there anything you’d like to talk about in relation to your treatment?

Version 2:

a) How did you hear about the trial?

a. Which treatment are you on?

b) Is this the treatment that you preferred?

b. b. …and with hindsight?

c) How long have you been on your treatment?

d) How did you feel after your first treatment?

e) What is your understanding of why you are taking this treatment?

f) Take us through what happened at your last visit to clinic?

g) How long does it does it take you to get to clinic?

h) What do you have to do at home for your treatment?

i) How has the treatment affected your daily life?

g) How is your quality of life since starting the trial?

k) Have you been referred to talk to anyone about your condition?

l) Do you have any new symptoms since taking the treatment?

m) Does your treatment affect your family/social life?

n) Do you have pain? Has this been helped by the bisphosphonates?

o) Is there anything you’d like to talk about in relation to your treatment?

Sample size estimates in qualitative research interviews are more art than science and depend on the quality of the data collected. For this study, data collection ceased at the discretion of each researcher, which coincided with our target group sizes. As experienced researchers and interviewers, we were also mindful of inappropriate impositions on this patient population.

The researchers digitally recorded the interviews and wrote field notes, where appropriate (with the participant’s permission), to record incidents occurring during the interview, non-verbal communication or reactions at the time of the interview. The interviews were transcribed in full, and verbatim and anonymised transcripts were coded using NVivo 8 software. Standard operating procedures for data protection ensured participant details were secure at all times.

### Data analysis

The analytic framework for this qualitative sub-study is based on IPA [7,9]. This approach is increasingly used to address health-care and quality of life research topics, where the aim is to understand the meaning that events or states have for participants based on their subjective accounts [10]. It is also interpretative in the sense that the researchers’ conceptions and experience, as brought to the analysis, are also recognized in a 'two-stage interpretation process, or double hermeneutic’ [10].

IPA is based on an idiographic approach beginning with a single case as a basis for developing more general categories in a detailed case-by-case analysis. The transcripts from the three sites were systematically analysed in several stages [8] as follows:

•Preliminary reading: The first transcript is read line by line and annotated with initial comments.

•Early analysis: Initial comments are grouped into themes.

•High-level abstraction: Connections between themes are developed until an organised master list and thematic account of the case is achieved.

•Subsequent transcripts: New themes are tested against the previous transcripts as non-recurring themes are tested against following transcripts. Connections across cases are noted to identify a set of superordinate themes for the group.

### Reflexivity and validity

Three interviewers were selected for their experience in the psychosocial issues of advanced cancer participants. All three interviewers (AN, DF and JM) have extensive clinical and academic experience of advanced cancer. DF and JM have made significant contributions to breast cancer research and teaching, and all three are familiar with a range of qualitative methodologies. In terms of interpretation, this level of experience adds understanding and depth to the analysis since the researchers were well aware of the illness trajectory and context of care, and these were the basis for further questions to individual participants. The interviewers clearly defined themselves as researchers and the interview time as a non-clinical event; however, patients were given information about how to access extra support if necessary at the end of the session. The data was analysed by a post-doctoral researcher supervised by AN and the results were discussed by the interview team at several meetings during the course of the study.

### Four superordinate themes emerged from the QUALZICE study interview data

1. Participants’ experience of the ZICE trial – to be presented here

2. Health-care professionals - the relationship between participants and clinical trial research nurses and doctors

3. Individual experience of illness – including the experience of being ill, self-image, hope and coping, and significant others

4. Interviewer’s actions - The data indicate that the three interviewers, who were involved in data collection, had somewhat different interviewing styles and ways of building rapport with the study participants. The literature on methodological issues related to interviewing cancer patients about their illness and treatment is currently somewhat scarce and thus an in-depth analysis of this data will lead to a potentially valuable methodologically oriented publication.

The purpose of this paper is to describe the experiences of people taking part in this trial and to describe differences between the two methods of treatment delivery. Therefore data from the first superordinate theme, which directly relates to the ZICE trial, will be reported here. Data captured within the remaining themes will be presented elsewhere.

## Results

### Participants’ experience of the ZICE trial

**Figure 1:**
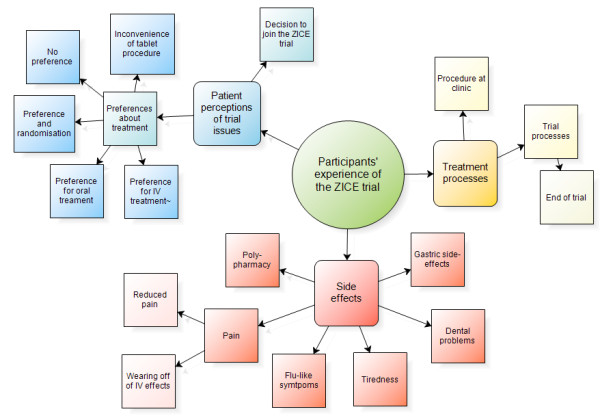
Participants’ experiences theme.

This theme captures participants’ experiences of the clinical trial and relates to topics concerning treatment and location as per the study aims, but also trial processes and procedures, and the impact of the trial assessments and treatment side effects. Figure 1 displays the theme and its associated subthemes.

### Theme 1: Patient perceptions of trial issues

Generally, participants who were interviewed in the QUALZICE study had a clear understanding of the randomisation process of the ZICE trial, which allocated them to either IV zoledronate at a clinic or oral ibandronate at home. They realized that neither they nor their doctor were able to choose the treatment they would receive:

*I didn’t have a choice*, *obviously the computer chose which*, *which one I was gonna go on*, *so I didn’t actually have a choice. (SU07*, *semi-urban area*, *IV treatment)*

However, there were cases where the participant was clearly interested in receiving the oral treatment and the trial was therefore perceived as a chance of getting that treatment. These quotations seem to indicate that some participants thought that they were on the trial only if they were receiving oral treatment:

*He [the doctor] said*, *'I don’t know whether I can get you on it*,*’ at that stage he had no idea*, *anyway*, *to cut a long story short*, *he did get me on the trial and that’s how it all came about. (R07*, *rural area*, *oral treatment)*

*We decided that rather than go ahead with the intra…*, *intravenous drip*, *we would* try *and get on the*, *on the trial*, *and fortunately*, *I did. (U06*, *urban area*, *oral treatment)*

#### Decision to join the trial

Participants reported three main reasons for joining the ZICE trial: the possibility of receiving the oral treatment, being constantly monitored and helping others. Some participants felt that participating in the trial, on either arm, would give them more security, as they were coming to the clinic more regularly and could therefore receive additional attention from the health-care professionals:

*For me it means that there’s always somebody checking up on me more*, *so I feel a bit more secure. (R11*, *rural area*, *IV treatment)*

Altruism was the third frequently mentioned reason for deciding to join the ZICE trial. Some participants felt that as they were supposed to receive the bisphosphonate treatment anyway, then being able to help others while doing so was an additional benefit. In some cases the philanthropic reasons were intertwined with a feeling of personal gain:

*I’m quite pleased to be in the trial and it works both ways*, *helping other people*, *but uh*, *it also means that I’m monitored on a regular basis*, *so*, *that*, *I don’t have a problem with that at all. (R03*, *rural area*, *IV treatment)*

#### Preferences about treatment

##### *Preferences for oral treatment*

Despite having a clear understanding of the randomisation process, the majority of interviewed participants had a clear preference about the treatment they were hoping to receive after randomisation. Most of those participants who had a clear initial preference were hoping to be randomised to the oral treatment. One of the reasons for wanting the oral treatment was the inconvenience of clinic visits:

*’Cos we live so far away from here [the local cancer centre]*, *I didn’t want to be*, *extra trips*, *coming up just to have a*, *an injection or*, *we’ve tried to tie it in with other treatment and it just seemed a bit*, *you know*, *lots of organisation. If you could take*, *take tablets? Yes*, *it’d be easier*, *just to take that at*, *that at home*, *so [laughs] all for that. [laughs] (R14*, *rural area*, *oral treatment*, *61 minutes travel to hospital)*

Some of those participants who preferred to receive the oral treatment considered the difficulties with intravenous treatments and discomfort with needles and blood that they had experienced previously, as the main reason for wanting tablets:

*Participant: It [oral treatment] was the one I wanted*, *without a doubt.*

Interviewer: Why was that then?

*Participant: Just because my veins are so bad and I have a bit of a phobia with needles as well. (U04*, *urban area*, *oral treatment)*

In addition to these practical reasons, some participants perceived the oral treatment as more normal and more effective. Because the oral treatment meant a reduced number of clinic visits, some participants saw it as creating fewer interruptions to their ordinary daily routine, thus allowing them to continue with their lives as normal. Others felt that if they did not have to go to the clinic they could almost forget about their illness and carry on with their lives, without being constantly reminded of their condition. For some participants the oral treatment felt more effective due to its continuous nature:

*I thought tablet would be*, *would be sort of more normal you can say*, *having it on a daily basis and less*, *sort of*, *dramatic*, *you know? (U02*, *urban area*, *oral treatment)*

*Well I thought logical sense*, *that if you actually had the steady drip of a drug that*, *into you every day*, *that it’s got to be more effective than having a big blast of it once a month and then it petering out towards the end of the month. (R09*, *rural area*, *oral treatment)*

##### *Preferences for IV treatment*

Although the preference for the oral treatment was expressed by more than half of all participants, a minority were hoping to receive the intravenous treatment, whilst a greater number were unsure or had no preference. Some participants had previously experienced difficulties with swallowing tablets; others were feeling sick as a side effect of their other treatments, thus making the oral treatment somewhat problematic:

*Somehow or other I preferred the infusion*, *you know*, *I thought to myself*, *'I prefer the infusion to the tablets*,*’ you know*,*’cos I imagine the tablets to be enormous and I’m not very good at swallowing*, *so*, *um*, *yes*, *I was quite happy with the infusion. (R08*, *rural area*, *IV treatment)*

Interestingly, many of those participants who preferred to receive the intravenous treatment referred to this as being a safer option. Because the intravenous treatment meant more clinic visits, the participants felt that they were better monitored and could receive additional attention from health-care professionals. Another reason for perceiving the intravenous treatment as a safer option was the reality of receiving it in the clinic, thus the treatment could not be missed or forgotten (although as a specific interview question, very few participants stated that they had missed oral doses). Receiving the intravenous treatment in the hospital environment and it being administered by medical professionals made it also seem more 'real’ than tablets, which would have been taken alone at home:

*I suppose*, *you don’t like being at the hospital*, *but actually*, *maybe they are sort of keeping a slightly closer eye on what’s happening if you’re in the system more frequently. That’s how I felt I think at the time. I don’t feel like that at the moment*, *but that’s how I think I felt at the time. [laughing] So maybe that was the plus side of it. (U16*, *urban area*, *IV treatment)*

##### *No preference*

In addition to those participants who had a clear preference about treatment, there were also those who claimed not to have had particular preferences. This indifferent or passive attitude was linked to faith in the care they were receiving and the fact that neither of the treatments created additional interruptions to their daily routines. Some participants also felt that they should be grateful for receiving a treatment at all, rather than complain about the possible inconveniences it might create:

*To be honest with you*, *if they had said the tablet*, *I had thought well*, *as long as uh*, *you know they’re giving me this*, *and I don’t care what I have*, *so long as I’m getting it. (U15*, *urban area*, *IV treatment)*

##### *Preferences and randomisation*

In the context of clinical trials, where participants are randomised to different treatment arms without taking their preferences into consideration, it is interesting to explore the reactions of those individuals who ended up receiving the treatment they did not wish to receive. Among the individuals who were interviewed in the QUALZICE study, there were nine participants who were not randomised to the treatment arm they initially preferred. Eight of those participants wanted to receive the oral treatment, but were randomised to the intravenous treatment.

Interestingly, among those nine individuals who were receiving the treatment they initially did not want to receive, there were only two who were disappointed with the treatment they were receiving. One of those participants, a woman who had received the intravenous treatment for almost three years, but wanted to receive the oral treatment, had asked to swap several times, but her request had always been refused:

*I hoped and prayed I wasn’t going to have the infusion*, *[laughs] and of course I ended up with the infusion. Um*, *only because it’s painful and um*, *I thought tablets were a damn sight easier*, *but it came up with the infusion and that’s it*, *you know*, *you’ve got to accept it haven’t you. (R01*, *rural area*, *IV treatment)*

The other participant who was disappointed was receiving the oral treatment. She had previously felt sick as a side effect from another treatment and thought that from that perspective the intravenous treatment would have been better for her:

*I would say probably I would have the injection if I thought that I was going to keep being sick and all of that*,*’cos that did worry me because I just think I*, *I don’t want to be missing out on this tablet’s not in my system. So perhaps the*, *perhaps the injection might have been better for me. (SU05*, *semi-urban area*, *oral treatment)*

The remaining seven individuals, who all had wanted the oral treatment but had been randomised to the intravenous treatment, were generally satisfied with their treatment. For some of them the initial preference for the oral treatment was linked to practical problems, such as discomfort related to intravenous treatments or the inconvenience of clinic visits, but once a solution to these issues was found, they were generally pleased:

*Um*, *probably the tablets*, *only because they had trouble with my veins*, *but that’s sorted now because I’m on the PICC [peripherally inserted central catheter] line so it’s no problem. (U16*, *urban area*, *IV treatment)*

##### *Inconvenience of tablet procedure*

Most participants who were on the oral treatment found the tablet procedure fitted well into their everyday routine and did not cause any significant inconvenience or interruptions:

*This treatment that – no*, *no*, *it’s just*, *just like taking tablet*, *no*, *easy*, *you know. (R14*, *rural area*, *oral treatment)*

The fact that participants could not eat or drink anything for at least six hours before taking the tablet did create some discomfort. For example, participants found this aspect of the tablet-taking procedure was difficult to follow in situations where they woke up with a headache. It was also difficult for those who could not sleep well and were used to having a drink during their sleepless hours. It is interesting to see how trivial but habitual aspects of participants’ everyday lives, like having a cup of tea in bed before getting up, suddenly acquired significance and are missed:

*The difficulties I suppose*, *if you can call them difficulties*, *um*, *is*, *say for example*, *I don’t sleep very well. Um*, *so*, *whereas perhaps I’d have got up*, *maybe had a cup of tea*, *read a magazine*, *gone back to bed. Ah*, *four hours later*, *five hours later*, *whatever – or two or three in some cases - um*, *got back up again*, *I can’t do that. But*, *ok*, *that’s*, *I can live with that. Um*, *the other thing*, *for example if*, *say you wake up in the morning and you’ve got a headache. And you can’t do anything about that until you’ve taken*, *until you’ve taken your tablet. … It’s about 30*, *40 minutes before you can do anything really*, *after the tablet. Or certainly that’s my understanding. … Um*, *so*, *I wouldn’t call them*, *really*, *disadvantages*, *for* me, *that far outweigh the minuses. (U06*, *urban area*, *oral treatment)*

The data also indicate that some participants had misinterpreted the information given in the participant information leaflets, interpreting some aspects more strictly than needed and remembering other aspects slightly differently. For example, one participant seemed to have interpreted the information about the need to stay in an upright position after taking the tablet as a need to stand up. At the same time she remembered that this 'standing-up period’ had to last for half an hour, though the participant information leaflet suggested staying upright for one hour. Another participant seemed to have understood that she had to refrain from eating or drinking for exactly six hours before taking the tablet:

*Well I hope I’m doing it right. Take it after*, *I’m just trying to think*, *six hours or something with no food*, *um*, *and you have to then be standing up so I call*, *call it my standing up pill*, *so um*, *so I have it first thing when I get out of bed in the morning and that’s the trouble you see when I have to stay standing up*, *so I have to work then. (R09*, *rural area*, *oral treatment)*

This extract illustrates the importance of information-giving but also checking understanding to ensure that participants are not subjected to undue inconvenience.

### Theme 2: Treatment processes

#### *Procedure at clinic*

The clinic procedure for participants on the ZICE trial seemed to follow a similar pattern in all three study sites. While participants were satisfied with the general procedure at clinic, they were dissatisfied with the extended waiting times in clinic:

*I go upstairs straight away and take my card. Ahhh I go*, *then get called to give some bloods*, *and then um*, *I go back into the waiting room and it’s about on a good day it’s about uh 20 minutes to half*, *but we get there*, *I have my blood taken*, *see the consultant*, *then I have to wile away two hours. The only complaint I’ve got is getting my treatment. There’s*, *the prescription is never ready so I always have to wait for the nurse to go to get the prescription because either the doctor doesn’t send it down or pharmacy are haven’t done it and they have to wait to get it checked. Quite often we’re there for a good two hours whereas my treatment only takes half an hour. (U03*, *urban area*, *IV treatment)*

#### Trial processes

The issue of informed consent about randomisation was discussed earlier together with participants’ decision to join the trial and their preferences for treatment. This section discusses participants’ understanding of their treatment, understanding of possible side effects and knowledge of the ZICE trial and procedures.

Participants’ understanding of the treatment was on most occasions correct. Most of the interviewed participants were aware that they were receiving bisphosphonate treatment to strengthen their bones:

*Well*, *I believe it strengthens bones*, *it uh puts*, *it was explained to me that it almost puts like cling film coating round the bones and protects it*, *I don’t*, *I don’t know if it stops it spreading*, *um I take Arimidex for the breast cancer*, *um to stop that spreading and um*, *do you know. I don’t know. (R01*, *rural area*, *IV treatment)*

However, while most of the participants were clear about the bone-strengthening effect of bisphosphonates, some participants were not clear:

*Interviewer: What*, *what’s your understanding of why you’re taking the tablet?*

*Participant: Not very much*, *quite frankly. I*, *I*, *I just feel that*, *uh*, *it’s a*, *a*, *obviously it’s a new drug fighting cancer. An’*, *and my particular type of cancer*, *the*, *uh*, *breast cancer of the bone. (R07*, *rural area*, *oral treatment)*

Most participants had an accurate understanding of the trial and its objectives. Participants understood that the trial aimed to compare the effectiveness of intravenous and oral bisphosphonate treatment:

*It’s er for the longer-term benefit of other people and obviously what proves to be the best treatment you know whether it’s this form or the tablet form erm that’s my understanding. (SU08*, *semi-urban area*, *IV treatment)*

Nevertheless, there were cases where participants had misinterpreted the information about the trial and its procedure. The aspect of the trial procedure that seemed to be most confusing seemed to be the length of the trial (see also below 'End of trial’):

*But I thought there were three parts to it? Wasn’t there a sort of long infusion*, *a tablet or a short infusion? I can’t remember*, *I think*, *what I understood was*, *the standard practice was an infusion that took longer than 15 minutes. That’s what I vaguely remember*, *that was what was*, *had been given in the past. (SU01*, *semi-urban area*, *IV treatment)*

*I’m not quite sure. Is it uh nine months*, *no? (U13*, *urban area*, *IV treatment)*

The issue of experiencing side effects was discussed in every interview and the findings related to this are reported below. Additionally, participants’ understanding and awareness about the possible side effects were discussed. The data reveal that participants’ views about side effects and how well they should be made aware of the possible side effects differed. Some participants felt that they wanted to be more informed and kept constantly aware of the possible side effects, whereas other preferred not to read the participant information leaflets:

*Every so often they should remind you actually*, *what the side effects are*, *what could be the side effects*, *you know. Because you go on it*, *you’ve got your bit of paper*, *um*, *you then*, *as I say if you get the tablet option*, *I have completely forgotten until you mentioned it. [laugh] Um*, *and then you get so caught up in pain*, *caught up in the spread of the cancer*, *that you kind of forget this other bit*, *and possibly the side effects of it. (SU01*, *semi-urban area*, *IV treatment)*

*To be honest*, *I tend not to read the side effects*, *because it ca—*, *I*, *it can*, *sort of*, *as you say*, *you know*, *it can frighten you a bit*, *can’t it? You*, *you got something that’s supposed to cure something that causes all these other things and you’re: 'Oooh*, *I don’t wanna know.’ (U06*, *urban area*, *oral treatment)*

##### *End of trial*

As mentioned above, participants seemed to be relatively unaware of the length of the trial, although this was clearly stated on the participant information sheet. Patients were followed up for 96 weeks, or nearly 2 years. Follow-up data was collected for a further three years but the patient did not have to attend for assessments. Perhaps, given the length of the trial it is not surprising that patients had forgotten some initial details.

Similarly, patients seemed not to have a very clear understanding of what is going to happen at the end of the trial:

Interviewer: Do you know how long you’ll be on the trial for? What’s your understanding of how long you’ll…

*Participant: Well I haven’t really been told for how long it is*, *I’m just assuming it’s for as long as I need it*, *I ju—*, *just don’t know. (U16*, *urban area*, *IV treatment)*

The participants who had come to the end of the trial had decided to continue with the trial drug indefinitely (as per trial protocol). Their doctors’ suggestion to continue seemed to be the main reason for their decision:

*Oh no*, *he just said that*, *um*, *that I’d come to the end of the trial and that I could take the drug if I wanted to take the drug*, *which I thought*, *well*, *what options are there*, *there’s no other options. (U09*, *urban area*, *oral treatment)*

The data thus indicate that participants’ ability to take in new information is limited and depends largely on their current interests and problems. While the information about the end of trial is important in the context of informed consent, clearly the processes related to the end of trial are not important for participants at the moment of joining the trial, and thus this information may not in reality play an important role in their decision-making. Reminding participants about certain trial procedures later in the course of trial, including the process of coming to the end of the trial, may be helpful.

### Theme 3: Side effects

Before giving an overview of the research findings related to side effects, it is worth reiterating that the aim of this study was not to collect data about the frequency of experiencing different side effects. Instead, the aim was to explore the experiences of participants receiving bisphosphonate treatment in two different forms – oral or intravenous. Hence, in the following we will discuss some of the side effects in relation to what it meant for the participants to have different side effects, in particular where this varied between the different forms, rather than a more detailed exploration of the frequency of side effects.

#### Polypharmacy

The participants who were interviewed in the QUALZICE study were typically receiving other treatments in addition to the bisphosphonate treatment. Herceptin and Arimidex were the most frequently mentioned other drugs received on prescription. Because of taking many different tablets or receiving several different intravenous treatments, the participants had difficulties with attributing certain side effects, which were collected via questionnaires in the main ZICE trial, to a specific treatment. Despite the interviewers’ efforts to understand whether the emergence of side effects coincided with the start of the bisphosphonate treatment, the participants in many cases still struggled to determine what part of their treatment was causing their side effects:

Tiredness. Fatigue. Nausea. And, um, you do something and you just can’t go. Or you do it and that’s your day ended…. It’s very difficult to say really’cos I’ve got pituitary problems and adrenalin problems … and I’m on, ah, steroid, which gives off the same things. So you’ve got two tablets doing similar things. And you’re not sure which one’s doing it. (U09, urban area, oral treatment)

#### Gastric side effects

As the data below indicate, participants on the ZICE trial reported gastric side effects, such as diarrhoea, indigestion, reflux and nausea. The sample size means that these results are descriptive rather than attributable to either trial arm and are presented in the context of participants’ daily lives.

In addition to creating general discomfort and pain, these symptoms also created interruptions to participants’ everyday activities, even if they were not experienced regularly or frequently. The participants who had experienced these symptoms once were more wary about their possible reoccurrence and thus avoided doing certain things that they used to enjoy doing (for example, going for a walk with a friend or eating certain food). These participants were also keener on staying at home to be able to deal with the symptoms in a convenient manner and avoid the symptoms occurring in a public place:

*I’ve had a touch of reflux*, *… I would*, *I would say it would be because of starting those tablets. I can’t*, *I don’t think it was to do with anything else that I can remember. (R06*, *rural area*, *oral treatment)*

The collected data reveal interesting differences in participants’ perceptions about the ways of dealing with gastric side effects. As the data below indicate, doctors prescribe stomach protectors, such as omeprazole, to participants who are experiencing gastric side effects. However, participants’ perception of whether stomach protectors can be used to ease the gastric side effects varies, with some participants not using the medicine as they believe that bisphosphonate tablets have to be taken on an empty stomach:

*I do get a bit*, *some nausea after taking it. And*, *um*, *if*, *if*, *if I leave it*, *I usually leave it an hour before I have breakfast an’*, *and it’s all clearer.*

*Well really that’s not*, *I take omeprazole. That*, *that isn’t really a help*, *because you’ve got to take the ibandronate on an empty stomach. … In*, *it can’t*, *it can’t take that*, *it*, *at least*, *well a minimum of half an hour after. (U12*, *urban area*, *oral treatment)*

#### Flu-like symptoms

Some of the participants who were receiving intravenous treatment reported having flu-like symptoms. These symptoms seemed to have occurred only after the first couple of treatments:

*Only on that initial one it felt very flu like and sort of achy erm but now I’ve got used to it so apart from obviously erm feeling a bit tired erm you know I don’t have any problems at all. (SU08*, *semi-urban area*, *IV treatment)*

#### Dental problems

In addition to generally checking for the understanding of possible side effects, some interviews focused specifically on the question about the possible dental side effects (osteonecrosis of the jaw). Interestingly, the bisphosphonate treatment did not create real dental problems for participants’, but it sometimes made it difficult for some of them to get the dental treatment that they needed:

*Like I had a problem couple of weeks ago – one of my crowns came off and they couldn’t fit it back. He did try but it came off again. He said*, *I can’t do it. He said normally*, *I take the tooth out*, *but I knew that if I had the tooth out I’d come off the um trial so in the end*, *he just left it there*, *he said he’d ground it down and he said I’ll just leave the root in. Now I wasn’t quite sure about that but I went yesterday and [the research nurse] was saying*, *oh yes that’s fine. She was quite happy with it if the dentist was so. (U14*, *urban area*, *oral treatment)*

#### Pain

Talking about pain was one of the recurring themes in the QUALZICE study. In this section, those parts of the participants’ interviews that referred to the pain they were experiencing in the present moment, that is, during the QUALZICE study, is discussed.

##### *Reduced pain*

The participants reported that the pain they experienced before starting their current treatment was on many occasions unbearable and debilitating, but that their pain levels had decreased significantly since starting their treatment. Because most participants were receiving different treatments simultaneously, it was difficult to determine which one was the most effective for pain control. Some participants attributed the decrease or absence of pain to the bisphosphonate treatment they were receiving. However, it was more common for participants to attribute the decrease in pain levels to the radiotherapy they had received, or to think that the combination of radiotherapy and bisphosphonate treatment was keeping their pain under control:

Interviewer: So when you did take the bisphosphonates that helped?

*Participant: Yes completely. No pain since. (U04*, *urban area*, *oral treatment)*

*Interviewer: So you wouldn’t say there’d been any change in pain*, *you haven’t had much pain before the treatment*, *that changed once you started the trial?*

*Participant: No*, *no*,*’cause I*, *I*,*’cos I had obviously [words incomprehensible] pain in the left foot and they zapped it with radiotherapy and that’s been so much better. I don’t really get a lot of pain at all. (R10*, *rural area*, *oral treatment)*

##### *Wearing-off effect of IV treatment*

As already mentioned, not all participants attributed the decrease in their pain levels to the bisphosphonate treatment. Nevertheless, several participants who were receiving intravenous treatment reported that they could feel their pain levels increase in the final week of their four-weekly bisphosphonate treatment. Because the participants were experiencing this wearing-off effect, it appears that some doctors had changed their treatment from four-weekly to three-weekly. However, this was not done for all participants who reported feeling more pain in the fourth week of their treatment cycle:

*Once I’ve had my um treatment I’m fantastic*, *you know*, *for like two*, *oh it’s brilliant*, *… But then the third week*, *like now this week*, *it’s this I’m waiting for next week to go*, *I start to ache – my body is kind of like trying to tell me I’m ready for it. … But once I have had it*, *within a day or so*, *I’m like a little button. (U01*, *urban area*, *IV treatment)*

#### Tiredness

Some participants complained about feeling tired and without energy. For some participants who mentioned this tiredness seemed to be related to the intravenous treatment; they felt tired the next day after getting the infusion. For others, tiredness seemed to be a general symptom, not specifically linked with the bisphosphonate treatment. The transitory fatigue associated with the bisphosphonate treatment seemed almost normal and was mentioned in passing. When it was perceived to be a more ongoing symptom then it was explained in a lengthier manner:

*Yeah*, *I think I felt tired. Um*, *but on the whole I didn’t feel any*, *any sort of serious effects. … But looking at the note*, *I mean the original thing I had about it*, *it seems to be one of the side effects anyway*, *doesn’t it? (R03*, *rural area*, *IV treatment)*

*I do feel tired after it. (U05*, *urban area*, *IV treatment)*

## Discussion

Participants’ reasons for joining the trial were largely philanthropic, as reported elsewhere [11], but they also reported joining for the chance of receiving oral medications (as IV was the standard treatment available outside the trial) and to receive additional attention from health-care professionals, which resonates with earlier work by Sulmasy et al. [12], who found that a primary motivation for trial participants was for enhanced treatment, with some leanings towards therapeutic misconceptions [13,14].

Participants were, on the whole, satisfied with the trial and their place within the trial process. There was, however, an underlying element in that participants seem to have interpreted that they were only in the trial by virtue of the treatment allocated to them. Hence, they thought they were getting a new treatment that was on trial, which they couldn’t otherwise have. This attitude has implications in relation to trial equipoise and both patients’ and medical teams’ understanding of the concept in the context of a clinical trial. Most participants who had a clear initial preference were hoping to be randomised to the oral treatment; this preference was expressed due to the inconvenience of clinic visits or difficulties with intravenous treatments and discomfort with needles and blood. Several participants reported needle phobia. Many participants reported that the insertion of needle into their veins had become increasingly difficult, as could be expected for this participant group. Certain chemotherapy drugs have a vesicant effect and may cause long-term damage. Repeated venepuncture is also associated with scarring and sclerosis in cancer patients, whose skin may already be delicate. Participant experience of this issue will further inform the main trial outcomes and the analysis of trial attrition rates due to this reason. This is a lengthy trial for those participants who may become venous compromised, and it raises questions concerning the management of this issue. Nevertheless, the findings show that most participants dealt with the intravenous drug administration well and that, once practical difficulties were overcome, there were advantages with this route.

On the other hand, although the oral route had been the route of choice for many, there were disadvantages as well as advantages. The taking of daily medication was a reminder of the cancer and the restrictions imposed on eating caused disruption to some people’s lives.

The findings also point to the complex issue of providing and seeking information on treatment options. On the one hand patients need to be, and may want to be, thoroughly informed about the options available to them [15,16]; on the other hand they may choose to ignore the information provided or make sense of it in ways that contradict or disregard the medical experts’ advice [17,18]. Thus, there are challenges for those who provide care for cancer patients, for they have to find a balance between participants’ need to make sense of their own situation and also correctly and sufficiently informing them of the reality of their condition.

Despite rigorously developed trial information documents and research nurse support, participants frequently appeared uninformed about procedures, for example, the reason for blood tests, end of trial procedures and the oral treatment protocol. It appears that participants’ ability to take in new information is limited and depends largely on their current interests and problems. While the information about the end of trial is important in the context of informed consent, clearly the processes related to the end of trial are not important for participants at the moment of joining the trial, and thus this information may not in reality play an important role in their decision-making. Reminding participants about certain trial procedures later in the course of a trial, including the process of coming to the end of the trial, may thus be necessary. Furthermore, given some participants’ misunderstandings about the oral protocol and end of trial arrangements, the importance of information-giving and also checking understanding to ensure that participants are not subjected to undue inconvenience is highlighted. This emphasises the need for research nurses to ensure participant understanding is evident at all stages of the trial process.

The bisphosphonate treatment did not create significant dental problems for participants, although several reported extra visits to their dentist with one participant attending appointments every six weeks. The fact that participants were on bisphosphonate treatment did make it difficult for some of them to get the dental treatment that they needed. Some dentists were reportedly wary of participants receiving treatment and referred them elsewhere.

### Strengths and limitations

The key strength of this paper is that it reports on patient experiences of the treatment arms, as an outcome measure for the main ZICE trial, based on interviews with patients currently participating in an ongoing clinical trial of an investigative medicinal product. Qualitative studies are extremely rare in this context.

The sample comprises six homogeneous groups. This is a large sample for an IPA study but as with any qualitative study, the results are not generalisable nor do they make any theoretical contributions to the social science literature. Instead they complement the main trial findings and the reported outcomes for the patient group receiving these treatments.

## Conclusions

Geographical characteristics relating to the differences between urban and rural areas were not an issue for participants due to the trade-off between distance and the time taken to travel through heavy traffic.

Participants frequently referred to the oral treatment in terms of its acceptability as more normal and more effective. They also mentioned that this format was more 'continuous’ in controlling symptoms. For those participants randomised to receive oral treatments on the ZICE trial, the fasting and upright protocol was well tolerated by most but misunderstood by several, who were reluctant to use their prescribed stomach protector medicines before taking the tablet. One participant referred to it as her 'standing-up tablet’, as she thought she needed to be on her feet for the protocol. Most participants missed their early morning cup of tea. Several participants on the intravenous arm complained of a wearing-off effect.

The main practical reason for preferring the intravenous treatment was participants’ previously experienced difficulty with taking tablets. Participants reported that the pain they experienced before starting their trial treatment was on many occasions unbearable and debilitating, but that their pain levels decreased significantly following the start of the trial treatment.

In summary, the recommendations, for consolidation in future studies of this type, from this study include:

•Qualitative interviews are well received by clinical trial participants. This approach is more efficient and less costly if embedded in the full trial design.

•Consider the practical difficulties faced by people undergoing intravenous infusions and offer suitable alternatives, for example a PICC, injection at their community health clinic rather than going to the hospital or even that the district nurse comes to their home.

•Check participant comprehension of trial interventions and processes at multiple time points throughout the trial.

•Provide participant education and ensure understanding of pain control and opiate use.

•Consider a participant-held information card in the context of dental care.

The main ZICE trial aimed to assess the non-inferiority of either medication. However, participants’ views regarding the differing modes of delivery should not be assumed. This study has shown that in a trial context, participants’ views can usefully add to the main trial outcomes, especially in a trial of non-inferiority. The ZICE trial has reported that ibandronate failed to satisfy non-inferiority criteria in terms of the skeletal-related event rate in bone-metastatic breast cancer compared with zoledronic acid, and this should be considered along with patients’ views when prescribing in the real world.

## End note

^a^Main ZICE trial ref numbers: Eudract No: (2005-001710-40).

## Abbreviations

IMP: Investigational medicinal products; IPA: Interpretative phenomenological analysis; IV: Intravenous; NCRI: National Cancer research Institute; PICC: Peripherally inserted central catheter; TMG: Trial management group; WCTU: Wales cancer trials unit.

## Competing interests

The authors declare that they have no competing interests.

## Authors’ contributions

AN contributed to the study design,data analysis, and manuscript. DF and JM collected and analysed data. CS and EH contributed to the data analysis and manuscript preparation. NM was the co-chief investigator. DW was the principal investigator and Trial Management Group member. KH contributed to the study concept and gave a critical review. GG is data custodian and gave a critical review. PBL was the chief investigator and gave a critical review. All authors read and approved the final manuscript.
